# Altered Resting-State Functional Activity in Patients With Autism Spectrum Disorder: A Quantitative Meta-Analysis

**DOI:** 10.3389/fneur.2018.00556

**Published:** 2018-07-24

**Authors:** Wenhui Wang, Jia Liu, Shaojie Shi, Taiyuan Liu, Lun Ma, Xiaoyue Ma, Jie Tian, Qiyong Gong, Meiyun Wang

**Affiliations:** ^1^Department of Radiology, Zhengzhou University People's Hospital and Henan Provincial People's Hospital, Zhengzhou, China; ^2^Department of Radiology, Union Hospital of Tongji Medical College, Huazhong University of Science and Technology, Wuhan, China; ^3^School of Life Science and Technology, Xidian University, Xi'an, China; ^4^Institute of Automation, Chinese Academy of Sciences, Beijing, China; ^5^Huaxi MR Research Center, Department of Radiology, West China Hospital of Sichuan University, Chengdu, China

**Keywords:** autism spectrum disorder, meta-analysis, regional homogeneity, functional neuroimaging, resting state

## Abstract

**Background:** There is accumulating evidence showing that patients with autism spectrum disorder (ASD) have obvious changes in resting-state functional brain activity. So far, there have been no meta-analyses of the resting-state brain activity alterations in patients with ASD. We attempted to explore the resting-state functional activity changes in patients with ASD, possibly providing a new perspective for investigating the pathophysiology of patients with ASD.

**Methods:** We screened relevant studies published before August 2017 in PubMed, Ovid, Web of Science, China National Knowledge Infrastructure (CNKI), and the Wan-fang database. Fifteen resting-state functional neural activity datasets (including 382 patients and 348 healthy controls) were included. Through the use of the effect-size signed differential mapping (ES-SDM) method, we carried out a meta-analysis of resting-state functional activity studies of patients with ASD.

**Results:** Compared with healthy controls, patients with ASD showed hyperactivity in the right supplementary motor area, middle frontal gyrus, inferior frontal gyrus, the left precentral gyrus, and the bilateral cerebellum hemispheric lobule (VIII/IX), and hypoactivity in the right middle temporal gyrus, superior temporal gyrus, and the left precuneus, posterior cingulate cortex, median cingulate cortex, and bilateral cerebellum (crus I).

**Conclusion:** This meta-analysis indicates that patients with ASD have significant and robust resting-state brain activity alterations in the language comprehension network, inferior-posterior cerebellum, default mode network (DMN), and cerebellar crus I. These brain regions may serve as specific regions of interest for further studies of ASD, which will allow us to further clarify the neurobiological mechanisms in patients with ASD.

## Introduction

The main clinical symptoms of autism spectrum disorder (ASD) include impairments in social communication and interaction, as well as patterns of stereotypic and repetitive behaviors (1). Although the neuropathology of ASD is still not clear, there is a broad consensus that abnormal activity in related brain regions plays a very important role in the neurodevelopmental disorder of autism.

The resting state refers to the state during which a subject is not performing an explicit task. In addition, resting-state brain activity, also known as the default mode network, is observed through changes in blood flow in the brain that creates what is referred to as the blood oxygen level-dependent (BOLD) signal, which can be measured using functional magnetic resonance imaging (fMRI). Because brain activity is intrinsic, which is present even in the absence of an externally prompted task, any brain region will have spontaneous fluctuations in the BOLD signal (2–4). Resting-state neuroimaging, which monitors intrinsic brain activity, has been proven to be a popular and reliable research tool that can provide important insights into the pathophysiology of ASD (5). So far, multiple neuroimaging modalities, such as fMRI, single-photon emission computed tomography (SPECT), and positron emission tomography (PET), have been used to investigate brain activity alterations in patients with ASD. In the previous resting-state studies, participants were asked to close their eyes and not perform any explicit tasks during acquisition of the data. In general, fMRI depends on BOLD signal fluctuations to show patterns of brain activity. Roughly two analytical methods have been used to quantify fMRI resting-state brain activity, including regional homogeneity (ReHo) and the amplitude of low-frequency fluctuations (ALFF). In addition, ASL-fMRI/PET/SPECT monitors brain activity alterations by means of cerebral blood flow (CBF) measurements. The three different techniques, ReHo, ALFF, and CBF, reflect local spontaneous neuronal activity in a similar manner.

Many of the previous studies showed resting-state neuronal activity abnormalities in the cerebral cortex and limbic regions, mainly including the thalamus, inferior frontal gyrus, right superior temporal gyrus, medial frontal gyrus, middle/posterior cingulate cortex, and cerebellum, in patients with ASD (6, 7). However, because of the interstudy differences in the clinical symptoms of the participants, the sample sizes, and the technical methods used for the data acquisition and analysis, the detailed results were different. Along with the increasing number of published studies of resting-state neuronal activity in patients with ASD, we attempted to carry out a meta-analysis to identify common abnormalities.

As a new meta-analytic technique, ES-SDM is a method based on the use of voxel coordinates to identify resting-state brain activity abnormalities in the whole brain rather than within discrete brain regions that have been widely applied to neuroimaging studies of several diseases, such as depression (8, 9) and migraine(10). However, there have been no meta-analyses of resting-state neuronal activity studies in patients with ASD using ES-SDM. Therefore, we tried to conduct a meta-analysis of resting-state brain activity studies to provide a path to understand the pathological underpinnings of patients with ASD.

## Materials and methods

### Study selection

We followed the Preferred Reporting Items for Systematic Reviews and Meta-Analyses (PRISMA) guidelines (11). We performed systematic and comprehensive searches in PubMed, Ovid, Web of Science, China National Knowledge Infrastructure (CNKI), and the Wan-fang database. Correlative studies were published from January 1985 to August 2017. The search keywords were as follows: (1) “autism” < OR > “Asperger” < OR > “ASD”; (2) “rest” < OR > “resting state”; and (3) ReHo < or > CBF < or > ALFF < or > ASL < or > PET < or > SPECT < or > amplitude of low-frequency fluctuations < or > regional homogeneity < or > cerebral blood flow < or > positron emission tomography < or > single-photon emission computed tomography < or > arterial spin labeling < or > neuroimaging.

We selected the included studies according to the following inclusion criteria: (1) autistic disorder or Asperger's disorder was diagnosed on the basis of the Autism Diagnostic Observation Schedule (ADOS), the Diagnostic and Statistical Manual of Mental Disorders-Fourth Edition (DSM-IV), or the Autism Diagnostic Interview-Revised (ADI-R) diagnostic criteria; (2) comparisons of patients with ASD with healthy controls over the whole brain; (3) original studies published in English or Chinese; (4) the whole-brain results shown in three-dimensional coordinates (*x, y*, and *z*) in the standard stereotactic space (Talairach or MNI); and (5) use of the ReHo or CBF or ALFF method at the whole-brain level. Studies were excluded according to the following exclusion criteria: (1) any other meta-analytical studies or literature reviews; (2) sufficient data for the meta-analysis were unavailable from the original study or after contacting the authors; (3) studies using the seed voxel-based analysis method or using region-of-interest (ROI)-based methods.

### Quality assessment

The quality of the included studies was assessed by a 10-point checklist that focused not only on methods for image acquisition and analysis but also on the demographic and clinical characteristics of the study participants. The checklist was based on previous meta-analytic studies (12, 13). The assessment details mainly included the diagnostic criteria applied, the sample size, the demographic and clinical characteristics, the imaging technique, the quality of the reported results, and the analytical technique (see Appendix, Table S1). Although the checklist may need to be improved, it still provided some objective indicators for the rigor of each study. The checklist requires that no fewer than two authors independently reviewed each paper and completed a full-scale rating. If the 2 ratings were inconsistent, after discussing the scores, a unified quality score was obtained. The final quality scores of the studies are shown in Table 1.

**Table 1:** Summary of 13 studies (15 data sets) included in the meta-analysis.

		**ASD patients**					**Healthy controls**			**Quality scores (out of 10)**
**Study**	**Modality/analysis**	**Diagnosis**	**Diagnostic tool**	**Age (M ± SD)**	**IQ (M ± SD)**	**N (F/M)**	**Age (M ± SD)**	**IQ (M ± SD)**	**N (F/M)**	
Dajani and Uddin (7)	rs-fMRI/ReHo	ASD ASP	DSM-IV	9.26 ± 1.28	112.44 ± 20.60	1/17	9.32 ± 1.35	112.72 ± 13.79	3/15	10
Dajani and Uddin (7)	rs-fMRI/ReHo	ASD ASP	DSM-IV	13.58 ± 1.86	104.55 ± 15.86	4/16	14.28 ± 1.78	104.95 ± 15.67	4/16	10
Dajani and Uddin (7)	rs-fMRI/ReHo	ASD ASP	DSM-IV	25.57 ± 6.32	108.53 ± 15.18	4/11	24.20 ± 4.16	111.07 ± 11.04	4/11	10
Maximo et al. (6)	rs-fMRI/ReHo	ASD	ADI-R and ADOS	13.8 ± 2.4	107.9 ± 19.0	4/25	13.5 ± 2.2	108 ± 8.9	7/22	10
Li (14)	rs-fMRI/ReHo	ASD	DSM-IV	12.14 ± 3.51	89.14 ± 9.21	22/28	12.01 ± 3.41	89.75 ± 9.37	20/30	10
Li et al. (15)	rs-fMRI/ReHo	ASD	DSM-IV and ADI-R	12 ± 3	83.87 ± 15.51	3/13	12 ± 3	91.06 ± 12.40	3/13	9.0
Paakki et al. (5)	rs-fMRI/ReHo	ASD	ICD-10	NA	NA	29	NA	NA	30	8.5
Wang (16)	rs-fMRI/ReHo	ASD	DSM-IV	3.4 ± 1.1	NA	4/16	3.3 ± 1.1	NA	5/15	9.0
Jann et al. (17)	ASL-fMRI/CBF	ASD	ADI-R and ADOS	13.8 ± 2.0	107.8 ± 18.7	4/13	12.8 ± 3.6	107.8 ± 14.3	3/19	10
Pagani et al. (18)	PET/CBF	ASD	ADOS-G and RAADS-R	31.8 ± 8.6	104.2 ± 17.1	6/7	28.5 ± 7.5	115.7 ± 10.8	5/5	9.5
Qiu (19)	SPECT/CBF	ASD	DSM-IV	2–11	NA	3/20	2–11	NA	2/8	9.0
Xiu (20)	SPECT/CBF	ASD	ICD-10	7.2 ± 3.0	NA	3/20	5.5 ± 2.4	NA	1/7	9.0
Itahashi et al. (21)	rs-fMRI/ALFF	ASD	DSM-IV	30.82 ± 7.39	105.60 ± 14.12	7/43	31.60 ± 7.60	108.09 ± 8.98	7/43	10
Cheng et al. (22)	rs-fMRI/ALFF	ASD	DSM-IV and ADI-R	4 ± 1	55 ± 14	0/33	4 ± 1	99 ± 23	0/26	9.5
Cao et al. (23)	rs-fMRI/ReHo/ALFF	ASD	DSM-IV	10.38 ± 1.39	89.27 ± 7.91	6/20	10.63 ± 1.13	92.75 ± 6.49	5/19	10

### Voxelwise meta-analysis

Meta-analytical group differences in resting-state functional brain activity between patients and controls were assessed using ES-SDM, a voxel-based meta-analytic approach (http://www.sdmproject.com/software). The SDM methods have been described in detail elsewhere (24–26) and are only described briefly in this study. First, we extracted the peak coordinates and effect sizes (e.g., *t*-values or *z*-scores) of all functional brain activity differences from each included study, which were statistically significant at the whole-brain level. Importantly, to avoid potential bias toward liberally thresholded regions, we ensured that each included study used the same statistical threshold. When only *z*-scores were reported in a study, an online converter (www.sdmproject.com/utilities/?show=Statistics) was used to convert the *z*-scores to *t*-values. Second, we used the peak coordinates and effect sizes of the group differences to recreate a standard Talairach map of the differences in ReHo, CBF, or ALFF for each study by means of a Gaussian kernel that assigned higher effect sizes to the voxels closer to the peaks. In the assignment, a relatively full-width at half-maximum (FWHM) was set at 20 mm to control for false-positive results (26). Unlike previous meta-analytic methods, such as the activation likelihood estimation (27) and multilevel kernel density analysis (28), both positive and negative coordinates were reconstructed in the same map, which prevented particular voxels from erroneously appearing to be positive and negative at the same time (24). Third, the mean map was obtained via voxelwise calculation of the random-effects mean of the study maps, weighted by the squared root of the sample size of each study, so that studies with large sample sizes contributed more. Finally, using standard randomization tests, we obtained statistical significance and then created null distributions from which the *p* values were obtained directly. The default ES-SDM kernel size and thresholds were used (full-width at half-maximum = 20 mm, voxel *p* = 0.005, peak height *Z* = 1, and cluster extent = 50 voxels) (26).

### Sensitivity analysis and heterogeneity analysis

We conducted a systematic voxel-based jackknife sensitivity analysis to test the robustness of the results from different studies at the whole-brain level. After discarding only one study each time, the rest of the studies were included to repeat the jackknife sensitivity analysis. Hence, the analysis was repeated 15 times. When a previously significant brain region was replicated in all or most of the included studies, the results were considered highly replicable and conclusive.

### Meta-regression of confounding biases

All the potentially confounding effects that may have influenced the analytic results, such as the mean age, gender ratios, and IQs of the patients and controls, were evaluated. We performed a simple linear regression of these factors to determine whether these factors influenced the meta-analytic resting-state brain activity results.

## Results

### Studies included in the meta-analyses

**Figure 1:**
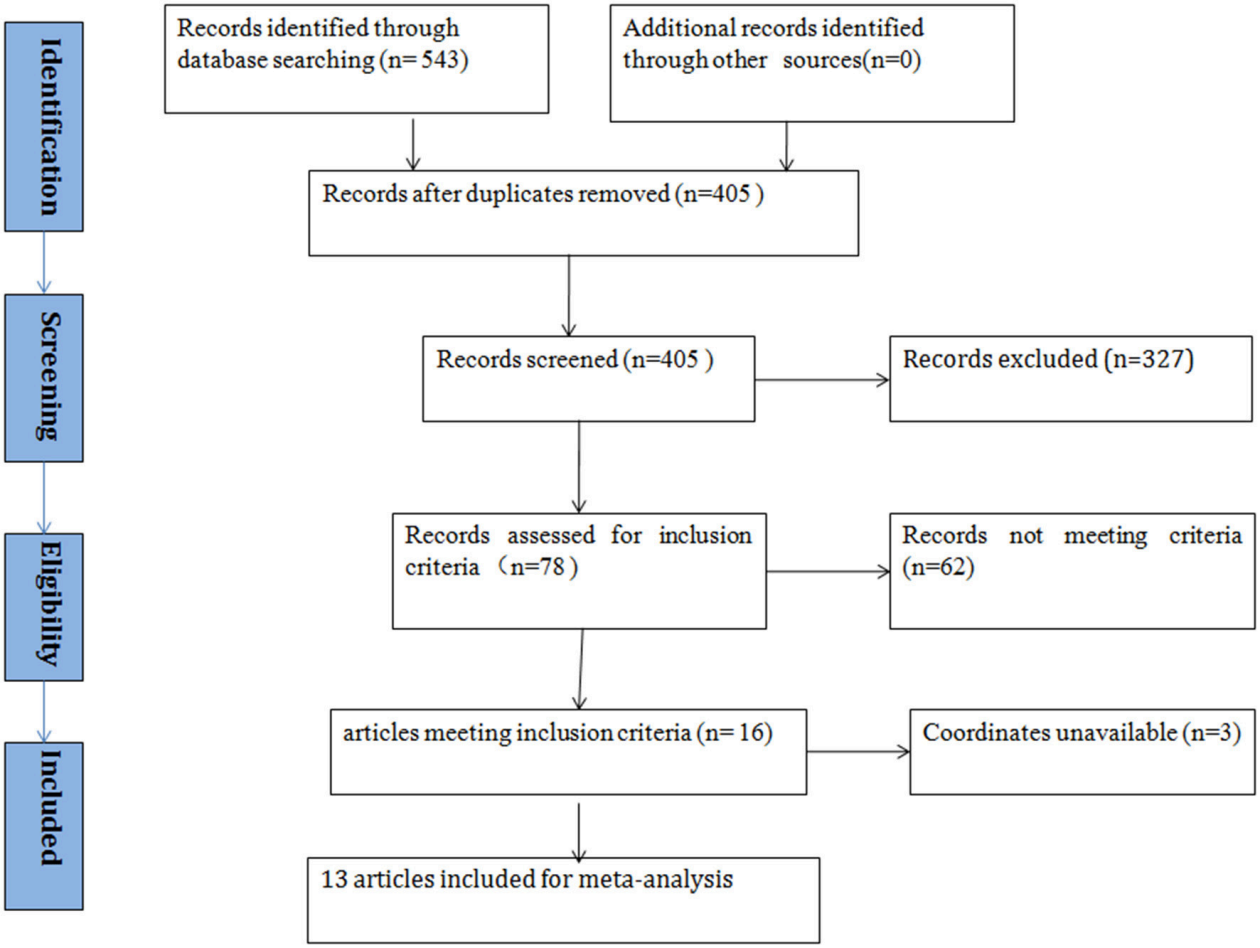
Meta-analysis of resting-state brain activity studies of patients with ASD.

According to the search strategy, we found a total of 405 studies. Ultimately, 13 studies met the inclusion criteria and were included in the ASD vs. healthy controls (HCs) comparison analysis. There were no additional eligible studies found in the reference lists. Participants were further stratified into Child, Adolescent, and Adult groups in one study, which was therefore treated as three unique studies. Thus, a total of 15 datasets were used to conduct the meta-analysis. Seven of the selected studies were written in Chinese and were translated into English for assessment, and the others were written in English. A flow diagram of the screening process is shown in Figure 1. In addition, some basic information about the subjects is presented in Table 1. In each study, there were no significant differences in age or sex between the patients with ASD and the healthy controls. However, the mean IQ of the healthy control group was marginally significantly higher than that of the ASD group.

### Meta-analysis of studies of ASD vs. HCs

**Figure 2:**
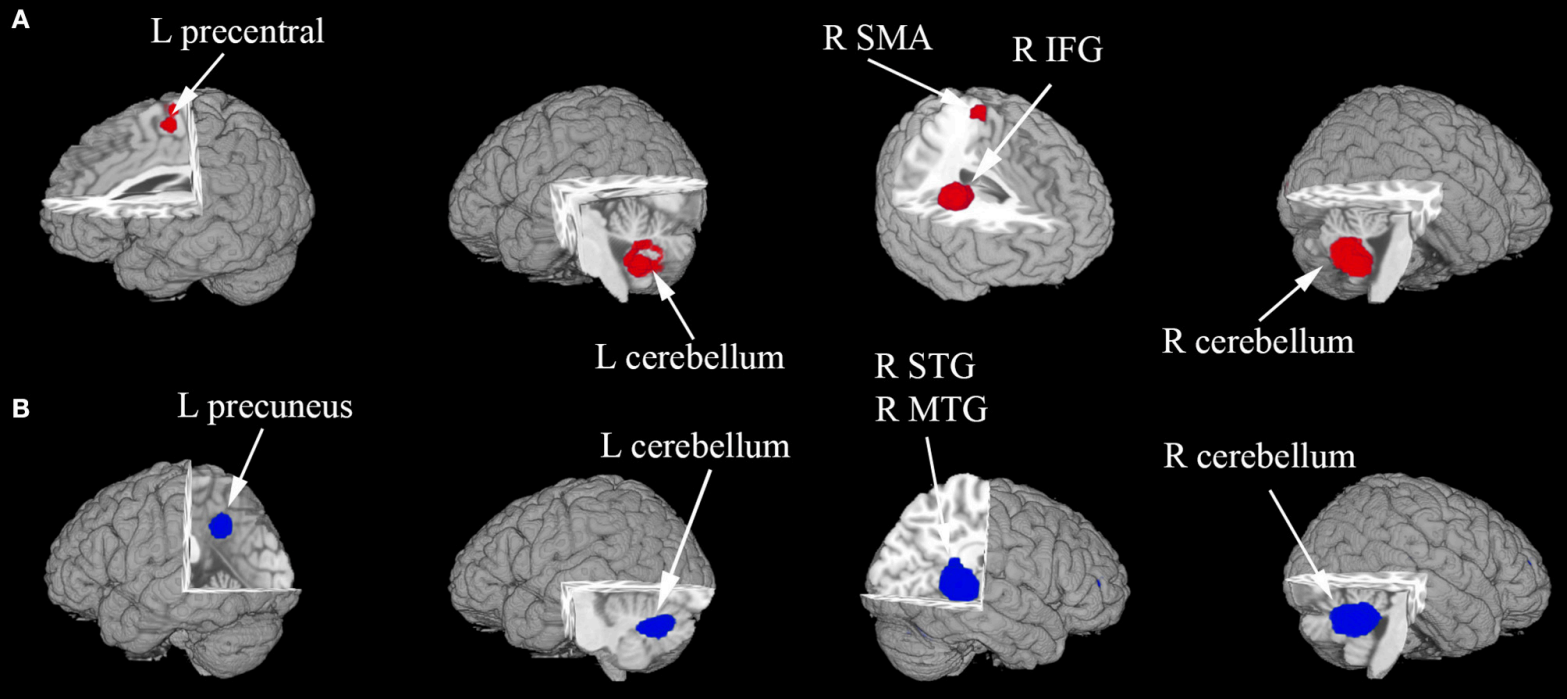
**(A,B)** The areas of decreased (blue) and increased (red) brain activity in patients with ASD compared with controls in the pooled meta-analysis. Altered resting-state brain activity in patients with ASD is displayed on a three-dimensionally rendered brain, with part of the left or right hemisphere removed. IFG, inferior frontal gyrus; L, left; MTG, middle temporal gyrus; R, right; SMA, supplementary motor area; STG, superior temporal gyrus.

In the pooled, whole-brain meta-analysis, compared with the HCs, resting-state activity was increased mainly in the right inferior frontal gyrus (IFG), middle frontal gyrus (MFG), supplementary motor area (SMA), left precentral gyrus, and bilateral cerebellum (hemispheric lobule IX, VIII) in patients with ASD. Compared with the healthy controls, the resting-state hypoactivity in the patients with ASD was mainly found in the right middle temporal gyrus (MTG), superior temporal gyrus (STG), left precuneus, posterior cingulate cortex (PCC), median cingulate cortex (MCC), and bilateral cerebellum (crus I) (Table 2, Figure 2).

**Table 2:** Brain regions showing greater and less activity in patients with ASD and healthy controls in the pooled meta-analysis (voxel-wise *p* < 0.005 and full-width at half-maximum 20 mm).

	**Maximum**			**Clusters**	
**Brain Regions**	**MNIcoordinates x, y, z**	**SDM value**	***p*-value**	**No. voxel**	**Breakdown (no. of voxels)**
**ASD** > **CONTROL**					
R supplementary motor area	8, −16, 72	1.399	0.001807332	58	R supplementary motor area, BA 6, BA 4(58)
R inferior/middle frontal gyrus	48, 12, 30	2.257	0.000014424	412	R inferior frontal gyrus, opercular part, BA 44, BA 48, triangular part, BA 48 (378) R middle frontal gyrus, BA 44, BA 45 (30) R precentral gyrus, BA 44 (4)
L precentral gyrus	−20, −14, 62	1.627	0.00055635	59	L precentral gyrus, BA 6(40) L paracentral lobule, BA 6(12) L superior frontal gyrus, dorsolateral, BA 6(6) L supplementary motor area, BA 6(1)
R cerebellum, hemispheric lobule IX	10, −58, −48	1.457	0.001313925	304	R cerebellum, hemispheric lobule IX(207) VIII(97)
L cerebellum, hemispheric lobule IX	−2,−54,−56	1.39	0.001901269	369	L cerebellum, hemispheric lobule IX(123) VIII(246)
**ASD < CONTROL**					
L precuneus, median cingulate / paracingulate gyri	−6, −48, 36	−2.207	0.00034678	276	L precuneus, BA 23(166) L median network, cingulum(41) L median cingulate / paracingulate gyri, BA 23(37) L posterior cingulate gyrus, BA 23(32)
R middle temporal gyrus	54,−44, 4	−2.632	0.000052631	595	R middle temporal gyrus, BA 22, BA 21, BA 42, BA 41(330) R superior temporal gyrus, BA 42, BA 22, BA 41, BA 21(263) R angular gyrus, BA 41(1) R supramarginal gyrus, BA 41(1)
R cerebellum, crus I	46, −54, −32	−2.158	0.000437617	493	R cerebellum, crus I, BA 37, BA 39,(439) II,(21) hemispheric lobule VI, BA 37, BA 19(15) R fusiform gyrus, BA 37(10) R inferior temporal gyrus, BA 37(8)
L cerebellum, crus I	−40, −70, −28	−2.048	0.000755548	284	L cerebellum, crus I, BA 19, BA 37,(275) II(1)
					L cerebellum, hemispheric lobule VI, BA 37, BA 19(8))

*BA, Brodmann area; L, Left; MNI, Montreal Neurological Institute; R, right; SDM, signed differential mapping*.

### Reliability analysis

The whole-brain jackknife sensitivity analysis of the ASD vs. HCs (Table 3) showed that the resting-state hyperactivity in the right IFG was highly replicable, as this finding was replicated across all 15 combinations of the datasets. The resting-state hyperactivity in the right SMA was also significant in all but one of the datasets. The resting-state hyperactivity in the left precentral gyrus and right cerebellum (hemispheric lobules IX and VIII) was also significant in all but two of the datasets. The resting-state hyperactivity in the left cerebellum (hemispheric lobules IX and VIII) was also significant in all but three of the datasets. The resting-state hypoactivity in the right MTG was highly replicated across all 15 combinations of the datasets. The resting-state hypoactivity in the right cerebellum (crus I), left precuneus, PCC, and MCC was significant in all but one of the datasets. The resting-state hypoactivity in the left cerebellum (crus I) was also significant in all but two of the datasets (Table 3).

**Table 3:** Sensitivity analyses of studies in the pooled meta-analysis of ASD vs. healthy controls.

	**Hyperactivation regions**				**Hypoactivation regions**				
**Discarded study**	**R IFG/MFG**	**Left precentral gyrus**	**R SMA**	**R cerebellum, hemispheric lobule IX**	**L cerebellum, hemispheric lobule IX**	**R MTG/STG**	**R cerebellum, crus I**	**L cerebellum, crus I**	**L precuneus/MCC/PCC**
Dajani and Uddin (7)	Y	Y	Y	Y	Y	Y	Y	Y	Y
Dajani and Uddin (7)	Y	Y	Y	Y	N	Y	Y	Y	Y
Dajani and Uddin (7)	Y	Y	N	Y	Y	Y	Y	N	Y
Maximo et al. (6)	Y	Y	Y	Y	Y	Y	Y	Y	Y
Cao et al. (23)	Y	Y	Y	Y	Y	Y	Y	N	Y
Li et al. (15)	Y	N	Y	Y	Y	Y	Y	Y	Y
Cheng et al. (22)	Y	Y	Y	N	N	Y	Y	Y	Y
Itahashi et al. (21)	Y	Y	Y	Y	Y	Y	Y	Y	Y
Jann et al. (17)	Y	Y	Y	Y	Y	Y	Y	Y	Y
Li (14)	Y	N	Y	Y	Y	Y	N	Y	N
Paakki et al. (5)	Y	Y	Y	N	N	Y	Y	Y	Y
Pagani et al. (18)	Y	Y	Y	Y	Y	Y	Y	Y	Y
Qiu (19)	Y	Y	Y	Y	Y	Y	Y	Y	Y
Wang (16)	Y	Y	Y	Y	Y	Y	Y	Y	Y
Xiu (20)	Y	Y	Y	Y	Y	Y	Y	Y	Y

*IFG, inferior frontal gyrus; L, left; MCC, median cingulate gyri; MFG, middle frontal gyrus; MTG, middle temporal gyrus; N, no; PCC, posterior cingulate gyrus; R, right; SMA, supplementary motor area; STG, superior temporal gyrus; Y, yes*.

### Meta-regression

The mean age, sex ratio, and IQ were used as factors of influence to conduct a regression analysis. In the meta-regression analysis of the studies of ASD vs. HC, no effects of mean age, sex ratio, or IQ were detected.

## Discussion

As far as we know, this whole-brain, voxelwise meta-analysis, which evaluated resting-state brain activity anomalies in patients with ASD compared with healthy controls, is original. Our results revealed that the most robust resting-state brain activity was increased in the language comprehension network (29, 30) and in the inferior-posterior cerebellum (31), which are composed of the frontal cortex and cerebellar hemispheric lobules (VIII/IX), respectively. Meanwhile, the most significant regions of hypoactivation were found in the default mode network (DMN) and cerebellar crus I. The findings were powerful and reliable according to the jackknife sensitivity analysis. There were no detectable effects of the mean age, sex ratio, or IQ on resting-state brain activity in patients with ASD.

In this meta-analysis, increased resting-state brain activity was mainly observed in the right SMA, MFG, IFG, left precentral gyrus, and cerebellar hemispheric lobules (VIII/IX). Because of the prominent role of the cerebellum in motor functioning, its linguistic functions have been overlooked for a very long time (32). Currently, it is thought that the cerebellum is actively involved in language, cognition, and affective modulation (33). Hodge et al.'s study supported that the posterior lateral cerebellum was highly linked to the frontal cortex, including Broca's area, which together form the fronto-cortico cerebellar language circuits, and the abnormal development of language circuits was related to cognitive function and language impairments (31). An increasing amount of neuroimaging research has focused on the language comprehension networks, which play a significant role in underlying language comprehension, such as semantic understanding and integration of information (34), understanding vocabulary in context (35), and flexible application of pragmatics and grammar (36). In healthy controls, the language comprehension network is left-cerebral hemisphere dominant (37, 38). However, individuals with ASD tend to use right-cerebral hemisphere regions (39, 40). Recently, more studies have used resting-state fMRI (rs-fMRI) to assess abnormal connectivity in the language comprehension neural networks in individuals with ASD (41, 42). Previous research has shown that the compensatory neural networks for language comprehension include the supplementary motor regions of the right hemisphere and left precentral gyrus (30). In line with the findings of anatomic abnormalities, previous studies on individuals with ASD have shown that partial cerebral regions in the language comprehension network overlapped with the regions of increased gray matter. For example, Ecker et al. (43) found that the gray matter (GM) volumes of the dorsolateral prefrontal regions were significantly increased in individuals with ASD. This observation of structural abnormalities is compatible with the findings of functional alterations in resting-state brain activity.

In our study, decreased resting-state brain activity was observed mainly in the left precuneus, PCC, MCC and right MTG, right STG, and bilateral cerebellum (crus I). These brain regions overlap with the components of the DMN (44), which greatly contribute to autism spectrum traits (45, 46). As one of the major resting-state brain networks, the DMN plays an important role in performing emotional and social processes, self-referential thought, and theory of mind; therefore, the DMN has become a focus of research (47). Raichle et al. found that the DMN was active during the resting state (44); on the contrary, performing cognitive tasks induces decreased activation (deactivation) in the DMN. In addition, Kay Jann et al. found decreased resting brain functional connectivity in the precuneus/posterior cingulate cortex areas of the DMN in children with ASD (17). It is possible that all of these functional abnormalities are due to structural abnormalities. Yang et al. (48) found reduced GM volume in the temporal gyrus/cerebellum crus I in patients with ASD. He et al. (49) performed one study combining ReHo and structural MRI that showed that patients with Alzheimer's disease (AD) had decreased ReHo in the PCC/precuneus and atrophy in the PCC/precuneus region. Perhaps, decreased resting-state brain activity in ASD is accompanied by reduced GM volume in the DMN. These changes in GM likely play significant roles in establishing interregional information processing in the brain (50). In addition, an increasing amount of research has shown that cerebellar abnormalities are associated with autism and contribute to different higher order cortical networks, including frontoparietal networks, as well as the DMN. Allin et al. (51) indicated that the cerebellar crus I has functional connectivity with the DMN. Furthermore, Halko et al.'s study (52) demonstrated that, by mediating the activity of default regions, crus I/II participates in the DMN. In line with this anatomical evidence (53), the Stereotyped Behaviors and Restricted Interests scores in the population with ASD were found to be correlated with GM differences in the cerebellar crus I/II. There may be a close association between altered resting-state brain activity in the above-mentioned brain regions and neuropathological mechanisms that lead to the clinical symptoms in ASD.

## Limitations

The present meta-analytical study had some limitations. First, the study needed larger samples to increase the power of the analysis. Second, the accuracy of our voxelwise meta-analysis may have been limited because the accuracy was not derived from an original study rooted in raw statistical image but instead from published studies. Third, it is worth noting that different ages would cause different results. During the natural progression of autism, there are developmental changes in anatomy, and in cognitive function, and these changes may affect resting-state brain activity. Fourth, our study included both patients with autism and patients with Asperger's syndrome; however, different subtypes of the classical autistic spectrum may have different etiologies, which may have influenced the results of our findings. However, there were an insufficient number of studies to carry out these subgroup analyses. Finally, common artifacts, such as head motion and breathing effects, may have influenced the acquisition of neuroimaging data.

## Conclusion

Despite the limitations mentioned above, our voxelwise meta-analysis found decreased resting-state brain activity in the left precuneus, PCC and MCC, and in the right MTG, right STG, and bilateral cerebellar crus I, as well as increased resting-state brain activity in the right SMA, MFG, and IFG, the left precentral gyrus, and the bilateral cerebellum hemispheric lobule (VIII/IX) in patients with ASD by using the ES-SDM method. However, the meta-regression analysis suggested that the mean age, sex ratio, and IQ had no effects on resting-state brain activity, and further studies with larger samples and more accurate assessments of the ASD stages are necessary. There may be a close association between altered resting-state brain activity and impaired cognitive and affective function in these brain regions. These resting-state brain activity alterations, combined with demographic characteristics, may help us understand the neuropathological changes in the population with ASD.

## Author contributions

MW contributed to the conception of the study. WW, JL, TL, LM, XM, and SS contributed significantly to the analysis and manuscript preparation. WW and JL performed the data analyses and wrote the manuscript. JT and QG contributed to the interpretation and discussion of the results of the analyses.

### Conflict of interest statement

The authors declare that the research was conducted in the absence of any commercial or financial relationships that could be construed as a potential conflict of interest.
