# Supplementary material for: Altered Resting-State Functional Activity in Patients With Autism Spectrum Disorder: A Quantitative Meta-Analysis
Source: Front Neurol. 2018 Jul 24;9:556. doi: 10.3389/fneur.2018.00556 (PMC6066523; doi:10.3389/fneur.2018.00556)
Supplement: Supplementary file 1 [file Table_1.DOCX]

Table S1 Quality Assessment Checklist (When criteria were partially met, 0.5 points were assigned)

| **Category 1: Subjects** | Score (0/0.5/1) |
| --- | --- |
| 1. Patients evaluated prospectively; specific diagnostic criteria applied; demographic data reported.  2. Comparison subjects evaluated prospectively; psychiatric and medical illnesses excluded; demographic data reported.  3. Important confounds (e.g. age, gender, IQ) controlled either by stratification or statistically.  4. Sample size per group > 10. | |
| **Category 2: Methods for image acquisition and analysis** | |
| 5. Whole brain analysis automated with no a priori regional selection.  6. Coordinates reported in a standard space.  7. Imaging technique clearly enough described to be reproduced.  8. Measurements clearly enough described to be reproduced. | |
| **Category 3: Results and conclusions** | |
| 9. Statistical parameters provided for significant and important non-significant differences.  10. Conclusions consistent with results; limitations discussed. | |
| TOTAL /10 | |
